# Eruptive papules during efalizumab (anti-CD11a) therapy of psoriasis vulgaris: a case series

**DOI:** 10.1186/1471-5945-7-2

**Published:** 2007-02-26

**Authors:** Michelle A Lowes, Francesca Chamian, Maria V Abello, Craig Leonardi, Wolfgang Dummer, Kim Papp, James G Krueger

**Affiliations:** 1 Laboratory for Investigative Dermatology, The Rockefeller University, New York, New York, USA; 2 Central Dermatology, St Louis, Missouri, USA; 3 Genentech Inc., San Francisco, California, USA; 4 Probity Medical Research, Waterloo, Canada

## Abstract

**Background:**

Newer biological therapies for moderate-to-severe psoriasis are being used more frequently, but unexpected effects may occur.

**Case presentations:**

We present a group of 15 patients who developed inflammatory papules while on efalizumab therapy (Raptiva, Genentech Inc, anti-CD11a). Immunohistochemistry showed that there were increased CD11b^+^, CD11c^+ ^and iNOS^+ ^cells (myeloid leukocytes) in the papules, with relatively few CD3^+ ^T cells. While efalizumab caused a decreased expression of CD11a on T cells, other circulating leukocytes from patients receiving this therapy often showed increased CD11b and CD11c. In the setting of an additional stimulus such as skin trauma, this may predispose to increased trafficking into the skin using these alternative β2 integrins. In addition, there may be impaired immune synapse formation, limiting the development of these lesions to small papules. There is little evidence for these papular lesions being "allergic" in nature as there are few eosinophils on biopsy, and they respond to minimal or no therapy even if efalizumab is continued.

**Conclusion:**

We hypothesize that these papules may represent a unique type of "mechanistic" inflammatory reaction, seen only in the context of drug-induced CD11a blockade, and not during the natural disease process.

## Background

Newer biological agents have dramatically improved therapeutic options for patients with psoriasis vulgaris requiring systemic therapy. Curiously, despite our knowledge of the target antigen of these biologic therapies, there may be unknown or unexpected biological effects. Efalizumab (Raptiva, Genentech Inc) is an FDA-approved treatment for moderate-to-severe psoriasis vulgaris. Recent phase III randomized, double-blind, placebo controlled trials have shown that an excellent clinical result (Psoriasis Activity and Severity Index, PASI 75) is obtained by week 12 in approximately 30% of patients [1-3]. Efalizumab is a humanized monoclonal antibody to CD11a, one of the chains of the β2 integrin lymphocyte function-associated antigen (LFA)-1. LFA-1 binds to intercellular adhesion molecules (ICAMs), allowing leukocyte migration across endothelial membranes during inflammation. Efalizumab appears to block trafficking of leukocytes (particularly memory T cells) into sites of inflammation, leading to a peripheral lymphocytosis [4]. There is also a decrease in dendritic cells with efalizumab treatment [5]. However, efalizumab may have additional effects as the LFA-1/ICAM-1 interaction is also important in antigen presentation to T cells, and trafficking of T cells in the epidermis.

During clinical trials with efalizumab, we observed patients who developed a variable number of small, scattered erythematous papules during the treatment period. The lesions were initially recognized by one of the authors and were called "Papp's papules" by a number of dermatologists. These lesions resolved without additional treatment or with mild-to-moderate topical corticosteroid application, while efalizumab was continued. The relationship of these papular lesions to previously described eruptions that develop while on efalizumab is unclear. An advisory group of dermatologists described a clinical eruption termed "localized mild breakthrough" during the early stages of efalizumab therapy [6]. While these lesions may be papules, they have not been characterized by histology or for cellular composition by immunohistochemistry.

We present a series of patients that developed these papular eruptions during efalizumab therapy, and characterize this reaction histologically. To determine how leukocytes might travel into the skin during efalizumab therapy, we also analyzed integrin levels on circulating leukocytes. We suggest that these lesions represent a unique drug-induced "mechanistic" eruption that occurs during CD11a blockade, where leukocytes enter the skin using alternative integrins, and the number and array of leukocytes in cutaneous lesions may be distinct from those in "normal" inflammatory processes (when CD11a is functioning in its usual manner). In addition, blockade of CD11c and therefore LFA-1/ICAM-1 interaction in the immune synapse may prevent initial and sustained T cell activation and limit the development of these lesions to small papules. Importantly, there is no evidence that this is a conventional drug hypersensitivity or allergic process.

## Case presentations

We collected biopsies from 15 patients receiving 1–2 mg/kg/week efalizumab as part of several IRB-approved clinical trials in North America. Informed consent was obtained for participation in the trial by each center. Overall clinical status was determined (PASI score) and blood taken for complete blood count where possible. Patients were included if they had a lymphocytosis, which indicated they had therapeutic levels of the drug. Details of the patients are summarized in Table 1. Patients were not included if their eruptions appeared after ceasing treatment, which may be more indicative of disease relapse from therapy withdrawal.

**Table 1:** Details of patients with erythematous papules while on efalizumab therapy

**NO**	**PASI baseline**	**PASI D84**	**BX DAY**	**PASI at biopsy**
1	17.8	6	D70	4.2
2	12.2	3.4	D84	3.4
3	14.6	0.9	D42	0.9
4	12	4.8	D84	4.8
5	12.6	2.9	D28	9.5
6	23.6	12.4	D84	12.4
7	16.3	9.8	D84	9.8
8	15.4	12.8	D66	11.4
9	12.4	3	D28	9.4
10	28.1	15	D77	~15
11	27	4.9	D53	2.4
12	8	6	D63	ND
13	12	ND	D84	ND
14	20.5	13.8	D 28	33.8
15	60	30	D 28	ND

ND, not done

**Figure 1:**
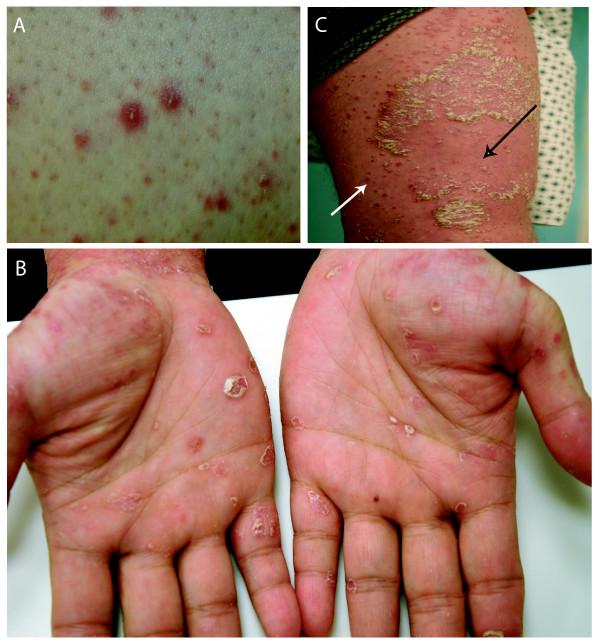
Clinical photographs from 3 patients with papular lesions while on efalizumab therapy, showing varied morphology of the papules. (A) Pustular lesions, (B) scaly lesions on the palms, and (C) erythematous lesions on the inner thigh (white arrow) adjacent to resolving plaque on the anterior thigh (black arrow).

Clinical photographs were obtained for several patients (Fig. 1), demonstrating a spectrum of presentation. There were erythematous lesions with variable scale, some of which appeared psoriasiform or occasionally pustular (Fig. 1A). The lesions were often in unusual locations such as the face or palms (Fig. 1B), scattered in flexural areas or extensor surfaces. They were more often in new areas, rather than areas with existing or previous psoriasis (Fig. 1C). At the time of the eruptions, all patients except one had improving psoriasis, with PASI scores well below starting levels. Central clearing of established plaques could sometimes be seen as these papules were emerging (Fig. 1C). Often, these papules developed toward the end of the treatment period. Some patients reported pruritis, while others were asymptomatic.

**Figure 2:**
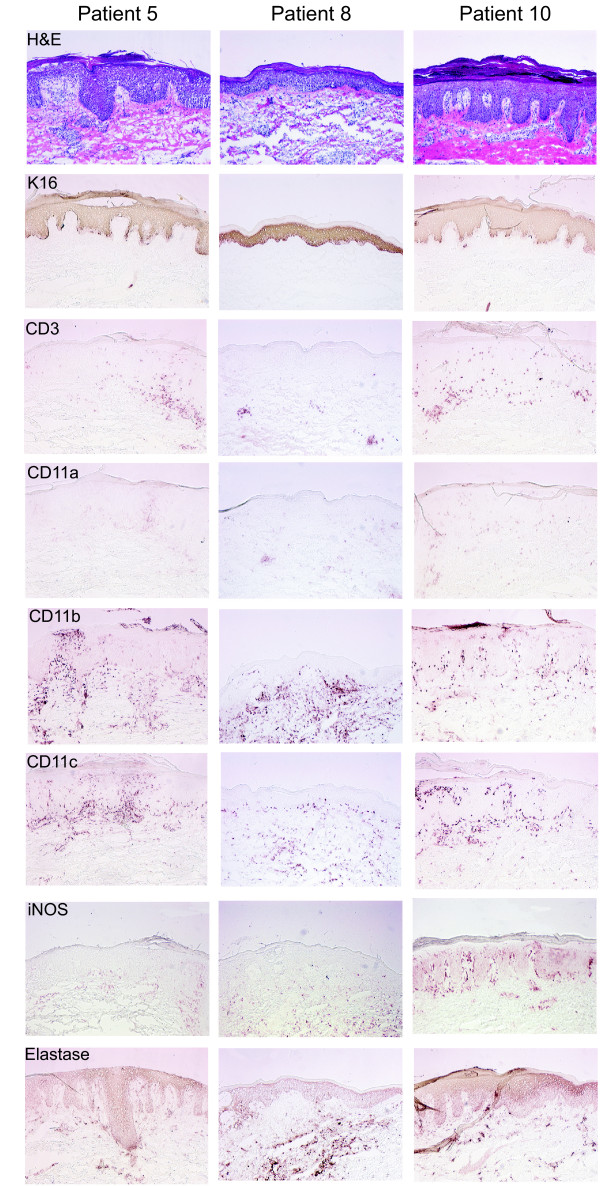
Immunohistochemistry of papular lesions from three representative patients. (A) Patient 5, (B) Patient 8, (C) Patient 10. Staining with H&E, keratin 16 (K16), CD3^+ ^T cells, α chain of β2 integrins CD11a (blocked by efalizumab), CD11b, CD11c, iNOS, and neutrophil elastase. There are abundant CD11b, CD11c^+^, and iNOS^+ ^cells in the lesions, with relatively less CD3^+ ^lymphocytes.

Immunohistochemistry was performed on papular skin biopsies to evaluate the leukocytic infiltrate of the lesions (Fig. 2), as well as hematoxylin (Fisher) and eosin (Shandon, Pittsburgh) staining. The following antibodies were used: keratin 16 (K16) (Sigma), CD3 (Becton Dickenson, BD), CD11a (Immunotech), CD11b (BD), CD11c (BD Pharmingen), iNOS (R&D Systems), elastase (BD), and DC-LAMP (Immunotech). In most lesions, there was some hyperkeratosis, occasionally alternating parakeratosis and orthokeratosis (only 1–2 cases), and there was epidermal hyperplasia, dermal edema and a mononuclear cell infiltrate. Where as normal epidermis is K16 negative, all lesions were K16 positive, indicating epidermal regenerative hyperplasia. The most striking observation was the large number of CD11b^+ ^and/or CD11c^+ ^leukocytes in both the epidermis and dermis. INOS positivity mirrored the CD11c^+ ^cell infiltrate. In contrast to untreated psoriasis, the infiltrate was relatively T cell poor, with scant neutrophil elastase staining, and few CD14^+ ^cells (not shown). While the lesions share some features of psoriasis, there are notably fewer T cells (e.g. especially Patient 8), fewer neutrophils, less epidermal acanthosis, and less consistent psoriasiform rete elongation. There was also a notable lack of organized dermal infiltrates consisting of T cells and dendritic cells.

CD11c^+ ^and iNOS^+ ^cells mark Tip-DCs, a new type of inflammatory dendritic cell which is present in psoriatic lesions [5]. We quantified the number of CD11c^+ ^and iNOS^+ ^cells in the papular lesions (n = 14), and compared these counts to normal skin (n = 10, n = 14 respectively) and psoriasis (n = 69, n = 10 respectively). The normal skin was obtained under an RU IRB-approved protocol from healthy volunteers, after obtaining informed consent. The CD11c^+ ^psoriatic lesional counts were from a large clinical trial with efalizumab, and this population has been previously described [5]. The iNOS counts in psoriasis lesional skin were also performed on pre-treatment biopsies from IRB-approved clinical trials in our department.

**Figure 3:**
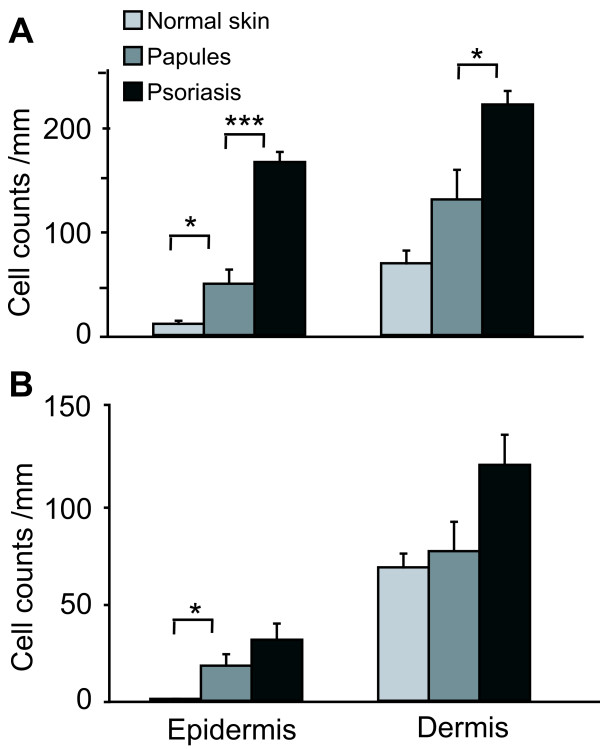
Cell counts for CD11c^+ ^and iNOS^+ ^leucocytes in normal skin, papular lesions, and psoriasis. There are intermediate numbers of (A) CD11c^+ ^and (B) iNOS^+ ^cells in the papules compared to normal skin and psoriasis. ANOVA with Dunnett's T3 test for multiplicity, * P < 0.05, *** P < 0.001.

We found that there were significantly greater numbers of CD11c^+ ^and iNOS^+ ^cells in the papular lesions compared to normal skin (Fig. 3), but intermediate levels compared to psoriasis. Cell counts in each group were analyzed by ANOVA. The three conditions (normal skin, papules, and psoriasis) showed differences in their mean values (p values for CD11c epidermis and dermis P < 0.0001, iNOS epidermis P = 0.002, dermis P = 0.009). For post hoc testing for multiplicity the Dunnett's T3 test was used. CD11c^+ ^cells were greater in the epidermis (mean 51 cells/mm) and dermis (mean 136 cells/mm) of papular lesions compared to normal skin (means 11 and 71 cells/mm respectively) (P = 0.042 comparing epidermal CD11c^+ ^cell counts in papules to normal skin) (Fig. 3A). The CD11c^+ ^cells were less than in the epidermis and dermis of psoriasis (mean 173 and 230 cells/mm respectively, (P < 0.0001 and P = 0.026 comparing papules and psoriasis epidermis and dermis, respectively).

INOS staining showed a similar pattern of intermediate numbers of positive cells in the papules, between normal skin and psoriasis (Fig. 3B). In normal skin, there are no iNOS positive cells in the epidermis, and dermal staining intensity is relatively low. In the papules there were significantly greater numbers of iNOS positive cells in the epidermis (20 cells/mm compared to 0 in the normal skin, P = 0.026). In the dermis, there were similar numbers of iNOS positive cells in papules compared to normal skin (80 and 71 cells/mm respectively, but again lower than psoriasis (126 cells/mm). Overall, there was infiltration of CD11c^+ ^cells into the dermis and epidermis of the papules, and iNOS production was increased, especially in the epidermis.

**Figure 4:**
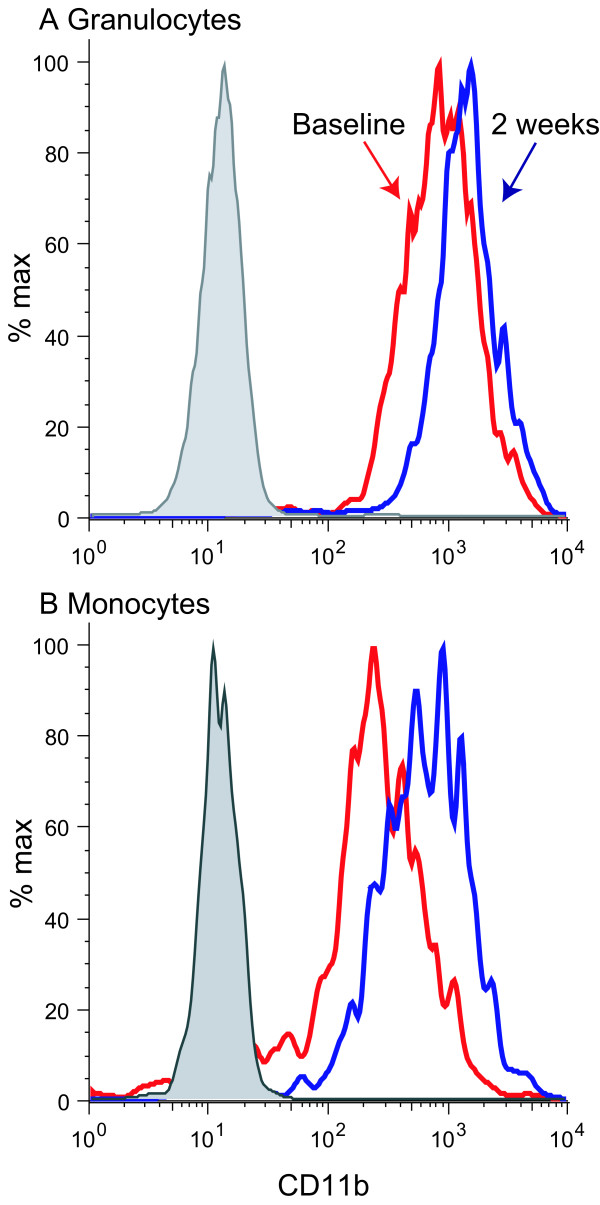
Levels of CD11b on leukocytes in patients receiving efalizumab therapy by flow cytometry. There is increased CD11b in some cases on (A) granulocytes and (B) monocytes, either with or without papules. Red line is baseline, blue line at 2 weeks treatment, shaded grey is isotype control.

As part of an ongoing study of mechanistic effects of efalizumab on leukocytes in psoriasis patients, we have studied expression of β2 integrins on circulating granulocytes, monocytes, and T cells. The following antibodies were used for staining: CD11a FITC (Immunotech), CD11b PE (BD) and CD11c PE (BD). Cells were acquired on the flow cytometer (BD FACS Calibur) and gated on cell populations by size or CD3 positivity. Some patients in this study, for example Patient 15 of this report, developed papules during treatment. In this patient we found increased expression of CD11b/CD18 on granulocytes (Fig. 4A) co-incident with the development of papules on the palms (Fig. 1B). CD11b was often increased on monocytes by 2 weeks of efalizumab treatment (Fig. 4B), although this was not restricted to patients with papules. There was an expected decrease in CD11a on all cells with saturation of the epitope by efalizumab in all leukocyte populations, and the mean fluorescence intensity decreased from baseline by greater than 50% [7]. CD11c showed small and variable changes on monocytes and granulocytes.

## Conclusion

Integrins are cell-surface heterodimers that mediate cell-cell and cell-matrix interactions [8]. The β_2 _group of integrins comprise a variable α chain (CD11a, CD11b and CD11c) and constant β_2 _(CD18) chain. CD11a/CD18, also called LFA-1, binds ICAM-1, -2 and -3, and is present on all leukocytes. As mentioned above, it has important roles in leukocyte trafficking across the endothelium, antigen presentation to T cells and immune synapse formation. These interactions are blocked by efalizumab. CD11b/CD18 (Mac-1) binds ICAM-1 and iC3b. It is present on myeloid cells, and some lymphocyte subsets. Therefore it has some similar functions to LFA-1. CD11c/CD18 (p150/95) binds fibrinogen and iC3b. CD11c is present on myeloid cells, and is a well recognized marker of myeloid dendritic cells and interstitial dendritic cells [9,10]. The β_1 _family, also called very late antigens (VLA1-6), comprise a series of molecules with a variable α chain, (CD49a-f) and constant β_1 _chain (CD29), and with a very wide pattern of expression. The VLA integrins are also involved in leukocyte trafficking.

Leukocyte emigration across the cutaneous vascular endothelium involves a series of steps mediated by various adhesion molecules in response to chemokine gradients: initial tethering of cells to the endothelium, loose rolling along the vascular surface, firm adhesion to the endothelium, and diapedesis between tightly apposing endothelial cells [11]. The cells then traverse the endothelial basal lamina and migrate through the extracellular matrix. Integrins are important for several of these processes, including tethering (VLA-4), adhesion (LFA-1, Mac-1, and VLA-4), and migration through the extracelluar matrix (Mac-1).

It is possible that in the absence of LFA-1 other integrins such as CD11b/CD18 or VLAs may allow leukocyte migration in response to inflammatory signals. For example, a CD11a knockout mouse model demonstrated aggravated Lyme carditis, [12]. Preformed CD11b is stored in leukocytes and rapidly upregulated on activation [13], and leukocyte CD11b upregulation has been seen in other settings of inflammation [13-15].

However, changes in circulating leukocyte integrins alone do not appear to be sufficient to induce these additional lesions. An external trigger may also be required, such as skin trauma, bacterial colonization, or microbial triggers, and then these activated cells are able to enter the skin using alternative integrins. Both CD11b^+ ^and CD11c^+ ^cells may be playing a role in the development of these papules. Increased numbers of CD11b^+ ^cells have been shown to be pathogenic in disease models such as autoimmune uveitis or lung infection [16,17]. Recently, we identified a population of CD11c^+ ^myeloid dendritic cells in psoriasis that produce TNF and iNOS, termed "Tip-DCs" [5]. These Tip-DCs were decreased with efalizumab therapy, in parallel with reduction in epidermal thickness. However, if CD11b^+ ^and CD11c^+ ^cells are able to traffic into the skin using alternative means, they may then release inflammatory mediators contributing to these papular lesions.

**Figure 5:**
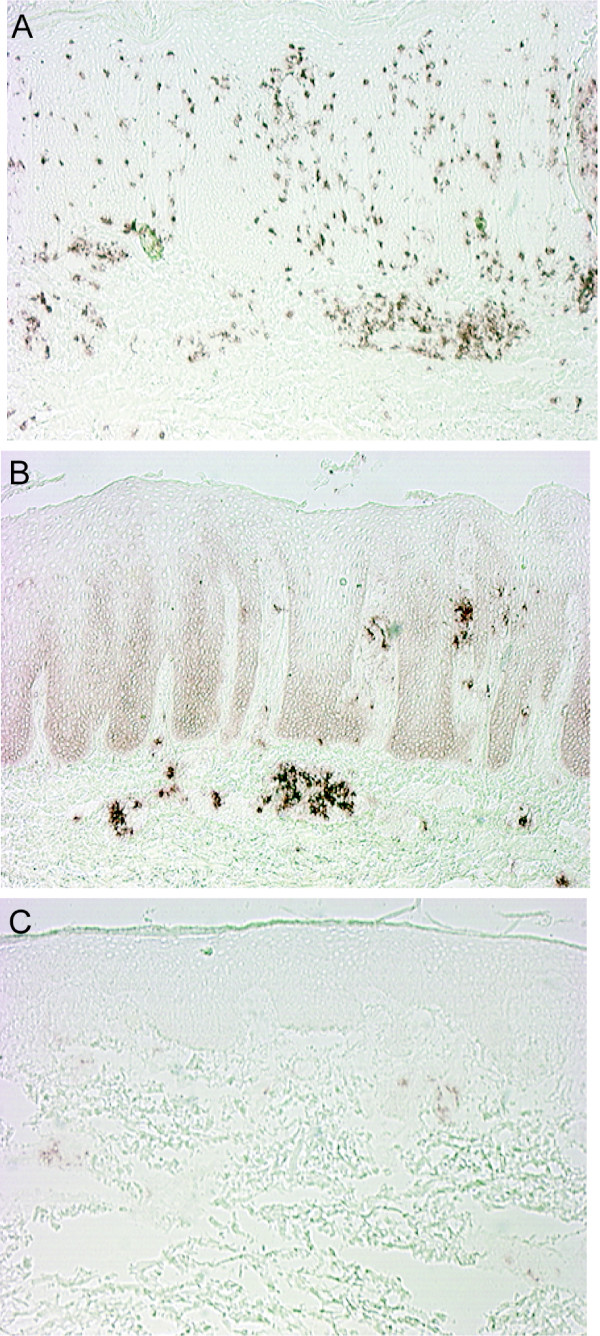
Decrease in DC-LAMP^+ ^dendritic cells in eruptive papules compared to psoriasis. (A) CD3^+ ^and (B) DC-LAMP^+ ^cells in psoriasis, showing abundant CD3^+ ^cells in the epidermis and dermis, and clusters of DC-LAMP^+ ^cells in the reticular dermis in lesional skin. (C) There is weak expression of DC-LAMP on only a few cells in a papular lesion.

While the above discussion is focused on alternative trafficking during CD11a blockade, the LFA-1/ICAM-1 interaction is also important for antigen presentation to T cells. This interaction forms the initial contact zone in an immune synapse between T cells and dendritic cells [18]. It is required to initiate T cell activation and may be crucial for the formation of a sustained immune response. In the dermis of mature psoriasis lesions, there are aggregates of T cells and DC-LAMP^+ ^mature dendritic cells creating secondary lymphoid structures [19] (Fig. 5A and 5B). The LFA-1/ICAM-1 interaction may allow formation of these aggregates, and in turn these T cell/dendritic cell aggregates may be responsible for the perpetuation and chronicity of psoriasis lesions. In these papules there is a notable lack of T cells and dendritic cells forming these organized dermal infiltrates. We found weak expression of DC-LAMP on few cells in a popular lesion (Fig. 5C). In this regard, these papular lesions could be considered an early or abortive stage in the formation of a psoriatic lesion. In the context of blockade of CD11a^+^, even if cells can use alternative means to enter the skin, the lack of CD11a prevents the LFA-1/ICAM interaction, and formation of dendritic cell/T cell aggregates, and thus the papules cannot proceed to a "full-blown" psoriasis lesion.

Recently, we described two psoriasis patients who experienced a flare of their disease while on efalizumab therapy [7]. Treatment was ceased at the time of the systemic flare, but was cautiously reintroduced at a later date, as there was no other therapeutic option. This is in contrast to the 5% of patients who develop a worsening of their psoriasis when therapy is ceased [20]. It is possible that both the small lesions presented here, flare on ceasing therapy, and the occasional systemic flare while on therapy may be part of the same spectrum. However, the smaller scattered lesions are clearly more common, and resolve without treatment, and a more significant additional stimulus may be required to cause a generalized eruption.

Importantly, there is no evidence of an allergic process in the development of these papular lesions: few eosinophils are seen on biopsy of these lesions, and they resolve with minimal treatment even if efalizumab therapy is continued. If this were an allergic reaction, continued therapy would cause worsening of the skin reaction, which is not the case.

The histological differences between these papules and psoriasis are that in the papular lesions there are reduced T cells, neutrophils, CD14^+ ^cells, lack of organized dermal T cell and dendritic cell aggregates, and the epidermal reaction has less acanthosis and psoriasiform rete elongation. We hypothesize that these lesions represent a unique event, a type of "mechanistic" eruption, seen only in the context of drug-induced CD11a blockade, and not during a natural disease process. We hypothesize that these lesions are unable to develop fully into psoriasis because T cell entry across the cutaneous vasculature, and T cell activation, are impaired during LFA-1/ICAM-1 blockade.

## Abbreviations

LFA-1 lymphocyte function-association antigen

ICAM-1 intercellular adhesion molecule

Tip-DC TNF- and iNOS-producing dendritic cell

iNOS inducible nitric oxide synthase

VLA very late anitgen

K16 keratin 16

PASI Psoriasis Activity and Severity Index

## Competing interests

WD is an employee of Genentech. CL, KP and JGK have served as consultants for Genentech Inc and Serono and received research support. The other authors do not have any financial interest related to this work.

## Authors' contributions

MAL, JGK wrote the manuscript and analyzed the data; FC and MVA performed research and analyzed data; CL and KP provided patient samples; WD organized the efalizumab study. All authors read and approved the final manuscript.

## Pre-publication history

The pre-publication history for this paper can be accessed here:


